# Current methods for development of rapid reviews about diagnostic tests: an international survey

**DOI:** 10.1186/s12874-020-01004-z

**Published:** 2020-05-13

**Authors:** Ingrid Arevalo-Rodriguez, Karen R. Steingart, Andrea C. Tricco, Barbara Nussbaumer-Streit, David Kaunelis, Pablo Alonso-Coello, Susan Baxter, Patrick M. Bossuyt, José Ignacio Emparanza, Javier Zamora

**Affiliations:** 1 Clinical Biostatistics Unit, Hospital Universitario Ramón y Cajal, IRYCIS, CIBER of Epidemiology and Public Health, Madrid, Spain; 2 Department of Clinical Sciences, Liverpool School of Tropical Medicine, Liverpool, UK; 3 Li Ka Shing Knowledge Institute, St. Michael’s Hospital, Unity Health Toronto, Toronto, Canada; 4 Epidemiology Division, Dalla Lana School of Public Health and Institute of Health Policy, Management and Evaluation at the University of Toronto, Toronto, Canada; 5 Queen’s Collaboration for Health Care Quality, Joanna Briggs Institute Centre of Excellence, Queen’s University, Kingston, Canada; 6 Cochrane Austria, Danube University Krems, Krems, Austria; 7 Canadian Agency for Drugs and Technologies in Health (CADTH), Ottawa, Canada; 8 Iberoamerican Cochrane Center-Servicio de Epidemiología Clínica y Salud Pública, Biomedical Research Institute (IIB-Sant Pau), Barcelona, Spain; 9 CIBER of Epidemiology and Public Health, Barcelona, Spain; 10 School of Health and Related Research (ScHARR), University of Sheffield, Sheffield, UK; 11 Department of Clinical Epidemiology, Biostatistics and Bioinformatics, Amsterdam University Medical Centres, University of Amsterdam, Amsterdam, The Netherlands; 12 Clinical Epidemiology Unit, Hospital Universitario Donostia, BioDonostia, CIBER of Epidemiology and Public Health, San Sebastian, Spain; 13 Barts and the London School of Medicine and Dentistry, Queen Mary University, London, UK

**Keywords:** Rapid reviews, Tests, Diagnosis, Knowledge synthesis, Decision-making

## Abstract

**Background:**

Rapid reviews (RRs) have emerged as an efficient alternative to time-consuming systematic reviews—they can help meet the demand for accelerated evidence synthesis to inform decision-making in healthcare. The synthesis of diagnostic evidence has important methodological challenges. Here, we performed an international survey to identify the current practice of producing RRs for diagnostic tests.

**Methods:**

We developed and administered an online survey inviting institutions that perform RRs of diagnostic tests from all over the world.

**Results:**

All participants (*N* = 25) reported the implementation of one or more methods to define the scope of the RR; however, only one strategy (defining a structured question) was used by ≥90% of participants. All participants used at least one methodological shortcut including the use of a previous review as a starting point (92%) and the use of limits on the search (96%). Parallelization and automation of review tasks were not extensively used (48 and 20%, respectively).

**Conclusion:**

Our survey indicates a greater use of shortcuts and limits for conducting diagnostic test RRs versus the results of a recent scoping review analyzing published RRs. Several shortcuts are used without knowing how their implementation affects the results of the evidence synthesis in the setting of diagnostic test reviews. Thus, a structured evaluation of the challenges and implications of the adoption of these RR methods is warranted.

## Background

Rapid reviews (RRs) have emerged as an efficient alternative to resource-intensive systematic reviews (SRs). RRs can speed up evidence synthesis by implementing methods and strategies to streamline the review process [1–4]. RRs can inform best practices for a diverse variety of clinical and public health topics requiring a quick turnaround [5–7]. Examples of topics where RRs are susceptible to be used to provide timely evidence include the identification of challenges to disease surveillance in the context of the crisis in Syria, the impact of e-health for rural residents in Australia and the adequate management of new emergent diseases as the COVID-19 disease [8–10].

The methods for performing systematic reviews are now well established for the field of medical test accuracy as with other areas of healthcare [11]. For diagnostic accuracy reviews, key characteristics include clearly-stated objectives and eligibility criteria; a systematic literature search; an assessment of methodological quality; and a systematic synthesis and presentation of the findings from the included studies [11–14]. In response to the demand for accelerated evidence syntheses to inform clinical decisions and policy, efforts have been made to standardize the methods and strategies for carrying out RRs while often extrapolating from effectiveness and safety RRs [4, 6, 7, 15, 16]. The RRs of diagnostic evidence however, present particular challenges given the fundamental differences between the methods used to summarize the evidence for interventions and those for diagnostic evidence. For instance, in contrast to SRs of interventions, SRs of diagnostic accuracy identify eligible studies from electronic search strategies that often involve screening thousands of titles and abstracts. The use of methodological filters can limit the volume of citations retrieved and is strongly discouraged [17, 18]. Moreover, in diagnostic accuracy SRs, the synthesis of evidence requires statistical knowledge to fit the complex statistical models needed for conducting meta-analyses [11, 12, 19].

In a previous scoping review, we examined the characteristics of RRs of diagnostic tests by scrutinizing repositories of Health Technology Assessment (HTA) agencies and papers published in indexed journals [20]. We found 191 RRs developed by international agencies since 2013—there was a clear increase better and more rapid synthesis of diagnostic evidence. We also observed that most RRs were broad in scope and assessed multiple index tests, outcomes, and test applications. We further found that well-known methodological tailoring strategies such as setting limits for literature searching by date, language, or number of databases were rarely reported. Due to an incomplete description of the methods used in the RRs, as well as inclusion of only published reports, we could not provide a detailed account of the current practice for the development of RRs of diagnostic tests [20]. To better understand how RR methods are currently used to synthesize diagnostic evidence, we performed an international survey to identify methodological practices used in the development of RRs for diagnostic tests.

## Methods

We developed and administered an online questionnaire seeking information about the methods and resources involved in the performance of RRs of diagnostic tests. We published a protocol summarizing the methods used to conduct this survey [21]. We followed the Checklist for Reporting Results of Internet E-Surveys (CHERRIES) guidance to report the findings of our research [22].

### Design of the survey

We developed and administered our questionnaire using SurveyMonkey software (https://surveymonkey.com/). We defined RR as “a knowledge synthesis strategy using limited or accelerated methods to expedite the time required to obtain a conclusive answer” [6]. In addition, we defined a diagnostic test as “any method for collecting additional information about the current or future health status of a patient” [23].

The questions in our survey focused on the methodology that responders use to conduct RRs, and drew on those elements that we previously identified in our scoping review [5, 20] including methods to:

- limit the scope of the review question (narrow the scope)
- focus on methodological tailoring of review steps according to the needs of decision-makers (review shortcuts)
- increase the intensity of the work on review processes (parallelization of tasks)
- use new technologies to fast-track selected review tasks (automation)

To gauge the level of experience and skills of the team, we included questions about the number of RRs of diagnostic tests previously conducted, the structure of the review team, and strategies for completing and publishing the final report.

We developed several drafts of the questionnaire prior to finalizing a pilot version. The pilot was tested by five researchers external to the research team who were asked to assess the usability and technical functionality of the survey. After this revision, the final version of the questionnaire consisted of 10 items: Six were multiple-choice questions, and four were open answers. A copy of the survey is available as a supplementary file.

### Recruitment process and description of the sample

On April 2019, we invited representatives from institutions around the world who perform evidence syntheses to participate in this closed survey. The invited representatives were from institutions belonging to the International Network of Agencies for Health Technology Assessment (INAHTA), the World Health Organization (WHO) collaborating centers on Health Technology Assessment (HTA), the Health Technology Assessment Network of the Americas (REDETSA), and the Health Technology Assessment International Network (HTAi, non-profit members).

All initial contacts were made by the principal investigator; participants were invited via an email message that included a personal invitation letter and a copy of the research protocol [21]. In addition, we provided a participant information sheet with details regarding the purpose of the study, reasons for their invitation, procedures involved in participation, as well as privacy and confidentiality processes. Participating institutions were asked to nominate a member of the team with experience in developing RRs to complete the survey.

Four reminders were sent by email to non-responders every 3 weeks until we closed the survey in July 2019. Participation in the survey was voluntary, and participants could leave the study at any time. No monetary incentives were offered.

### Survey administration

After receiving confirmation of participation in the research project, we sent an email containing the anonymized link to access the online questionnaire.

The questions in our survey were not randomized or alternated. Only the first question of the survey (Does your agency conduct rapid reviews (RRs) of diagnostic tests?) was mandatory; a negative answer to this question terminated the survey because the responder was deemed not to have the experience needed to answer the remaining questions.

Participants could review and modify their answers before submission using the back button. All responses were entered via the internet and automatically captured. Multiple entries from the same IP address were not allowed while the survey was open.

### Data analysis and ethical issues

This study was exempt from requiring approval by our Ethics Committee in accordance with Spanish National Regulations. A positive response to the invitation email was considered to be an agreement/informed consent to participate in the study.

Survey responses were de-identified and anonymized for all analyses. Duplicate entries were eliminated before analysis; only the final entry was kept for further analyses. Incomplete surveys were not included in the final analysis. We did not use the time spent to complete the survey as a criterion to exclude answers (questionnaires submitted with an atypical timestamp). We did not rely on statistical methods for assessing representativeness of the final sample. We performed all descriptive analysis using STATA 15.0.

## Results

Data were collected from April to July 2019. A total of 74 institutions were contacted by email, and we received 39 replies (53% response rate). The responders were located in Europe (20 agencies; 52%), America (11 agencies; 28%), Asia (4 agencies; 10%), Africa and Australia (2 agencies each one; 5% respectively). All but one of the institutions that had initially agreed to participate completed the survey (97% participation rate). The average time to complete the survey was around 11 min. Twenty-five (64%) of the 39 participants indicated that they performed RRs of diagnostic tests. They formed the final sample for our following analyses. The characteristics of participants (*n* = 25) are shown in Table 1.

**Table 1:** Characteristics of participating institutions

Country	N (%)
Africa	1 (4)
America	9 (36)
Asia	3 (12)
Europe	11 (44)
Oceania	1 (4)
**Number of RRs of diagnostic tests developed**	
Less than 10 RR	10 (40)
10 to 30 RR	9 (36)
More than 30 RR	6 (24)
**Availability of RR methodological guidance (i.e. handbook)**	
Yes	9 (36)
**Structure of RRs team**	
High level of training	22 (88)
Involvement of more than two reviewers or more than one team	10 (40)
Stakeholder involvement in several activities	10 (40)
**General methods of RRs**	
Development of a protocol	16 (64)
Use of a reporting template	12 (48)
Peer-review process	24 (96)
Public consultation of the draft review	4 (33)
Publication of the final review	11 (44)

The median number of RRs of diagnostic tests completed per institution at the time of the survey was 10 (Interquartile range from 5 to 18). Three institutions performed more than 100 RRs. Nine institutions indicated the use of a handbook or guideline to develop RRs (36%). Twenty-two institutions reported that these RRs are developed within a constrained time schedule (88%). In addition, the involvement of highly trained staff was a frequent element of RR conduct at participating institutions (88%). However, less than half of the institutions involved more than two reviewers or more than one team (i.e. to address different types of evidence, such as health outcomes and cost-effectiveness) during the performance of RRs (10 agencies; 40%). Ten agencies reported active participation of stakeholders during the development of RRs (40%; Table 1).

With regards to the general methods involved in the performance of RRs, 16 institutions developed and followed a protocol (64%). Most institutions reported a revision of the RR findings by an external and/or internal peer-review process (24 institutions; 96%). Only eleven institutions considered publishing the final RR (Table 1).

### Methods used to establish the scope of the review

All participants reported the implementation of one or more methods to pre-define the scope of a RR during the planning stage (range from 1 to 7 methods). A considerable number of participants reported implementing actions related to planning such as defining a structured PICO question (92%) and discussing the clinical pathway and the target condition with stakeholders (i.e., the role of the test in current practice and the intended applications) (68%). Only eleven participants reported limiting the number of outcomes (44%). All but one agency included accuracy as one of the main outcomes assessed in the development of RR of diagnostic tests (96%). Only one strategy was used by more than 90% of participants: defining a structured PICO question. One participant reported an additional method to narrow the scope of the RR: limiting the reference standards accepted for the target condition (Table 3).

### Methodological shortcuts

All participants reported the use of one or more methodological shortcuts when developing a RR of a diagnostic test (range from 4 to 13 methods) with six institutions reporting the use of up to five different shortcuts. The most common strategy was the use of a previous review as a starting point (e.g., to update the findings or to replicate the electronic searches; 92%) followed by limiting search strategies by language (e.g., to English only; 84%), and excluding additional sources of evidence such as conference proceedings (88%). Twenty-four participants reported using two or more limits when conducting search strategies (96%). In addition, nine agencies reported performing the screening, final selection, data abstraction, and quality appraisal with only one reviewer (36%) (Table 2).

**Table 2:** Rapid review methods reported by the survey participants

Method	N (%)
**Narrow the scope**	
Defining a structured PICO question	23 (92)
Discussing the clinical pathway for the target condition ^a^	17 (68)
Limiting the population ^b^	17 (68)
Limiting the number of index tests ^c^	19 (76)
Limiting the number of comparisons ^d^	14 (56)
Limiting the number of outcomes ^e^	11 (44)
Limiting the number of applications of the tests ^f^	20 (80)
**Review shortcuts**	
Using a previous review as a starting point ^g^	23 (92)
Limiting search strategies to one database	2 (8)
Limiting search strategies by language	21 (84)
Limiting search strategies by date	17 (68)
Limiting the syntax of search strategies ^h^	8 (32)
Limiting search strategies results using methodological filters	14 (56)
Excluding additional searches ^i^	22 (88)
Limiting screening of titles & abstracts: one reviewer only	12 (48)
Limiting the selection of full texts: one reviewer only	15 (60)
Limiting the data abstraction: one reviewer only	16 (64)
Limiting the quality appraisal: one reviewer only	10 (40)
Performing a narrative synthesis of findings ^j^	19 (76)
Excluding a GRADE assessment of findings	15 (60)
**Parallelization of tasks**	
Multiple reviewers completing the eligibility screening	8 (32)
Multiple reviewers completing the data abstraction	7 (28)
Multiple reviewers completing the quality appraisal	8 (32)
**Automation**	
Used to assist in the screening/selection of references	3 (12)
Used to assist in the data abstraction	2 (8)
Used to assist in the quality appraisal	1 (4)

Notes: ^a^ including the role of the test in the current clinical practice, its intended application, and prior/alternative tests; ^b^ ideally to one single population; ^c^ ideally to one single test; ^d^ ideally to one single comparison; ^e^ ideally to one single outcome; ^f^ ideally to one single application: i.e. monitoring, screening, diagnosis; ^g^ i.e. stepwise approach with an emphasis on higher levels of evidence, update of existing SR, re-run search strategies; ^h^, e.g. focused subject headings, terms in title only; ^i^, e.g. conference abstracts; search on the internet; ^j^ instead of a meta-analysis of data

An important number of participants did not perform a meta-analysis in RRs (76%) nor assessed the certainty of evidence (60%). Participants suggested two additional methodological shortcuts that were not included in our survey: partial verification of the screening, final selection, data extraction, and quality assessment by a second reviewer; and the choice of pre-existing evidence synthesis as the only eligible source of evidence (i.e. no primary studies are included) (Table 3).

**Table 3:** Additional rapid review methods reported by the survey participants

New methodologies		Comments
**Establishing the scope**	Limiting the accepted reference standards	Ideally to a single reference standard
**Limitations and shortcuts**	Selecting pre-existing synthesis of evidence only	e.g., systematic reviews, HTA reports
	Screening of titles & abstracts: selected verification	Two reviewers involved, checking a sample of/all references for accuracy
	Selection of full texts performed: selected verification	Two reviewers involved, checking a sample of/all references for accuracy
	Data abstraction: selected verification	Two reviewers involved, checking the sample/all references for accuracy
	Quality appraisal: selected verification	Two reviewers involved, checking the sample/all references for accuracy
**Parallelization and automation**	Multiple reviewers assessing the certainty of the evidence	i.e. using the GRADE approach
	Performing selected review activities simultaneously	e.g., data extraction of known studies while a search of new studies is conducted

### Parallelization of tasks

Twelve participants reported using one or more strategies to parallelize review tasks (48%) while three of these participants used all parallelization methods described in the survey. The two most frequently used strategies were the involvement of several reviewers in the screening of citations and quality appraisal (eight agencies; 32%) (Table 2). One participant reported that parallelization could also be used to assess the certainty of evidence using the GRADE approach. Another participant reported parallelization of review activities by performing selected activities simultaneously instead of consecutively (such as data extraction of known studies while a search of new studies is conducted) (Table 3).

### Automation of review tasks

Algorithms and machine learning techniques are methods rarely used by participating institutions. Only five participants reported the use of one or more of these techniques to perform RR tasks. Three agencies reported the use of software to assist in the screening and selection of references (11%; Table 2).

## Discussion

To the best of our knowledge, this is the first international survey assessing current practice of methods for diagnostic test RRs. With 25 participants from across all continents, we managed to generate a broad international sample. We obtained additional information about current strategies in use for development of these RRs in order to complement the findings from our previous scoping review [20].

Briefly, the general methods involved for the development of RR can be broadly classified into two groups: those limiting the scope and/or affecting the rigor of RR development; and those increasing the resources available for RR development [5, 6, 24, 25]. In the first group, we found that most strategies to narrow the scope are not used as a standard method; however, our survey indicated a greater usage of limits in the scope than our previous scoping review suggested [20]. In addition, we found a high number of participants imposing limits on the search, e.g., by limiting the language or date of electronic searches. We noticed that more than half of respondents use methodological filters during the literature search indicating that respondents are willing to potentially miss some studies in order to retrieve a manageable number of search results given the project’s shortened timeframe [17, 18]. We also confirmed that the participants often use a narrative synthesis to describe their findings rather than a formal data meta-analysis [20]. Participants also reported that they consider the inclusion of previous evidence synthesis to be useful for streamlining. While the use of pre-existing reviews was one strategy proposed for development of RRs, it is important to note that this strategy depends on the availability of existing SRs that satisfy the updated standards of preferred reporting items [26–28]. These may not exist for all topics.

Regarding the resources available for RR development, we found that a considerable number of institutions involved trained staff in the development of diagnostic RRs although there were usually only two reviewers involved. Selection and data abstraction by a single reviewer were common; however, it is possible that some institutions may prefer to perform selective verification by a second reviewer on a sample of the total citations. This strategy was suggested by the survey’s participants. We found that roughly one-third of institutions involved stakeholders in the development of RRs. While most standard SRs do not involve stakeholders in their production, RRs might be more relevant for decision-making in certain situations [29–31]. We further found limited use of task parallelization perhaps due to the lack of studies about the usability and impact of these strategies both in general and for diagnostic test RRs in particular [32–34].

Previous surveys of producers of knowledge syntheses reported slightly higher levels of adoption of RR methods compared to our findings [35]. These levels of adoption are also higher than those found in our previous scoping review. It is possible that RRs using methods similar to those used in SRs have a greater chance of being published [24, 36]. While we found that few RR methods are used by more than 90% of participants, we also observed that some SR tasks—such as developing a protocol and performing peer-review—are commonly implemented despite the time required for implementation. One possible explanation for this is that the extent of methodological modifications relies on a request from different stakeholders and therefore, in some cases, RRs can be produced following many of the same methods used in standard SRs [20, 37]. In addition, it is known that decision-makers are willing to accept only a small risk for an inaccurate answer in exchange for a rapid product; thus, current RR developers would be reluctant to compromise the validity of results in exchange for implementation of methodological shortcuts and limits [37, 38].

We acknowledge several potential limitations in this study. The descriptive goal of our research do not involve specialized statistical analysis. We obtained a 53% response rate from invited institutions based around the world; non-responders were located mainly in Europe and Latin America. Although we obtained replies from institutions based in similar locations, these missing data could have generated a risk of selection bias in our findings. We also found that 13 out of 39 institutions replying to our invitation do not conduct RRs and/or RRs of diagnostic tests. Also, participants in our survey were mainly representatives of local, national, and regional HTA agencies. The reports performed by these institutions might have characteristics that differ from other reviews (i.e. classic systematic reviews) produced in academic and research settings.

## Conclusions

To the best of our knowledge, this is the first international survey assessing the current practice of methods for diagnostic test RRs. Our survey indicates a greater use of shortcuts and limits versus our previous scoping review findings that was based on published RRs of diagnostic tests.

However, while our findings suggest that SR methods are sometimes preserved in the current practice of diagnostic test RRs, more general RR methods are usually applied to diagnostic evidence without a structured evaluation of the impact of their implementation. Nevertheless, due to the different characteristics of evidence synthesis used with diagnostic tests, there is a need to evaluate the commonly used methodological shortcuts specific to data sets comprising the RR on diagnostic tests as other researchers have claimed for their own fields [24]. In order to investigate this further, the next stage of our work is to conduct a series of interviews with experts in the diagnostic field to explore the potential challenges and implications of the adoption of these RR methods [21]. The findings of our research program on the RRs of diagnostic tests will be useful for the development of clear and tailored guidance in this field as well as to provide recommendations about adequate methods for rapid synthesis of diagnostic evidence for decision-making and policy development.

## Supplementary information

**Additional file 1.** Copy of online survey.

## Data Availability

The datasets used and/or analyzed during the current study are available from the corresponding author on reasonable request.

## Abbreviations

CHERRIES: Checklist for Reporting Results of Internet E-Surveys
GRADE: Grading of Recommendations Assessment, Development and Evaluation
HTA: Health Technology Assessment
INAHTA: International Network of Agencies for Health Technology Assessment
REDETSA: Health Technology Assessment Network of the Americas
RRs: Rapid reviews
SRs: Systematic reviews
WHO: World Health Organization
