# Supplementary material for: Current methods for development of rapid reviews about diagnostic tests: an international survey
Source: BMC Med Res Methodol. 2020 May 13;20:115. doi: 10.1186/s12874-020-01004-z (PMC7220561; doi:10.1186/s12874-020-01004-z)
Supplement: Supplementary file 1 — Additional file 1. Copy of online survey. [file 12874_2020_1004_MOESM1_ESM.pdf]

CHALLENGES FOR RAPID REVIEWS OF DIAGNOSTIC TESTS: AN INTERNATIONAL SURVEY

Invitation

We would like to invite you to participate in a survey to identify the methods currently used by rapid review developers to synthesise evidence on diagnostic tests. We have provided detailed information on the study in the following link (Participant information sheet) to help you decide whether or not you wish to take part. Please take time to read the information and feel free to contact us at any time in case anything is unclear. Participation in this survey is entirely voluntary.

Procedures

Completing the survey will take approximately 10 minutes of your time. We will ask questions about the methods and strategies currently employed by your institution to conduct rapid reviews on diagnostic tests. We are sure that the expertise of your team conducting reviews will contribute much to our research. We would really appreciate receiving your responses by June 25th-2019.

On behalf of the study team*,

Dr Ingrid Arevalo-Rodriguez (Principal Investigator)

Hospital Universitario Ramón y Cajal (IRYCIS), Madrid, Spain

* Researchers from Hospital Ramon y Cajal (Spain), Cochrane Austria-Danube University Krems (Austria), Li Ka Shing Knowledge Institute-St. Michael's Hospital (Canada), Dalla Lana School of Public Health (Canada), Cochrane Infectious Diseases Group-Liverpool School of Tropical Medicine (UK), Canadian Agency for Drugs and Technologies in Health (Canada), Iberoamerican Cochrane Center- Biomedical Research Institute (Spain) and Academic Medical Centre-University of Amsterdam (The Netherlands)

Rapid reviews of diagnostic tests

In our research, we defined **rapid review (RR)** as a knowledge synthesis strategy using limited or accelerated methods to expedite the time required to obtain a conclusive answer.

Also, we defined a **diagnostic test** as any method for collecting additional information about the current or future health status of a patient. Diagnostic tests include symptoms and signs, physical examination, laboratory and imaging strategies.

* Does your agency conduct rapid reviews (RR) of diagnostic tests?

- ◯ Yes
- ◯ No (End of survey)

Rapid reviews of diagnostic tests (continued)

In addition, we defined **accuracy** as the ability to correctly classify the presence or absence of a disease or a target condition

Accuracy is one of the outcomes assessed during the development of RR diagnostic tests in your institution

- ◯ Yes
- ◯ No

Rapid reviews of diagnostic tests (continued)

¿How many RR of diagnostic tests have been developed in your institution (approximately)?

1 50 100

For the following sections, please consider ***the three most recent rapid reviews of diagnostic tests*** developed by your institution.

In addition, please select the strategies and methods **commonly used** by your institution when a RR of a diagnostic test is conducted

A. Initial strategies in the conduction of rapid reviews of diagnostic tests

There are some general strategies that can be implemented to develop a rapid review, taking into account the needs of the stakeholder and available resources

Select all the general strategies used by your institution while conducting a rapid review of a diagnostic test:

- ☐ A protocol of the review is predefined and followed
- ☐ A formal PICO question is predefined and followed (i.e. population, index test, references standard)
- ☐ RR are developed within a constrained time schedule
- ☐ RR are developed by a highly trained team
- ☐ RR are developed by a large team (more than two authors)
- ☐ The stakeholder participates in several activities during the RR´s conduction

B. Mechanisms to enhance production of rapid reviews: Narrowing the scope

One of the mechanisms to enhance the timeliness of reviews is to limit the target population, interventions and outcomes considered in the rapid review

Select all the strategies used by your institution to limit the scope while conducting a RR of a diagnostic test

- [ ] The clinical pathway is predefined and discussed to help in narrowing the question
- [ ] The population is limited (mostly to one defined population)
- [ ] The number of index tests under assessment is limited (mostly to one test)
- [ ] The number of comparisons under assessment is limited (mostly one comparison)

B. Narrowing the scope (continuation)

Select all the strategies used by your institution to limit the scope while conducting a RR of a diagnostic test

- [ ] The number of assessed outcomes is limited (mostly to accuracy)
- [ ] The intended application of the test is limited (i.e. monitoring, screening, diagnosis)
- [ ] Other narrowing methods (please specify)

C. Mechanisms to enhance production of rapid reviews: Using review shortcuts

One additional group of mechanisms focuses on the reduction or omission of one or more systematic review steps

Select all the review shortcuts used by your institution while conducting a rapid review of a diagnostic test:

- [ ] Use a previous review as a starting point
- [ ] Search strategies are limited to one database
- [ ] Search strategies are limited by language
- [ ] Search strategies are limited by date (e.g. 5-10 years)
- [ ] Search strategies are limited using methodological filters
- [ ] Search strategies are limited by excluding additional searches (e.g. conference abstracts)

C. Using review shortcuts (continuation)

Select all the review shortcuts used by your institution while conducting a rapid review of a diagnostic test:

- [ ] The syntax of search strategies is limited (e.g. focused subject headings, terms in title only)
- [ ] Title & abstract are screened by one reviewer only
- [ ] Full texts are checked by one reviewer only
- [ ] Data abstraction is performed by one reviewer only
- [ ] Quality appraisal is conducted by one reviewer only
- [ ] A meta-analysis is not performed
- [ ] Other shortcut methods (please specify)

D. Mechanisms to enhance production of rapid reviews: Parallelisations of tasks

Other mechanisms focus on increasing the intensity of work on review processes, by splitting the workload and including multiple reviewers working simultaneously to complete review steps. Parallel tasks do not refer to core methods for performing standard systematic reviews, such as two reviewers working independently to select and assess studies

Select all the parallelisation strategies used by your institution while conducting a rapid review of a diagnostic test:

- [ ] Multiple people simultaneously complete the eligibility screening
- [ ] Multiple people simultaneously complete the data abstraction
- [ ] Multiple people simultaneously complete the quality appraisal
- [ ] Other parallelisation strategies (please specify)

E. Mechanisms to enhance production of rapid reviews: Automating review steps

Recently, automation algorithms (i.e. machine learning) have been proposed as useful methods to speed selected standard systematic review steps, for example, screening or data abstraction

Select all the automating review strategies used by your institution while conducting a rapid review of a diagnostic test:

- [ ] An automation algorithm is used to assist in the screening of references
- [ ] An automation algorithm is used to assist in the final selection of studies
- [ ] An automation algorithm is used to assist in the data abstraction
- [ ] An automation algorithm is used to assist in the quality appraisal
- [ ] Other automating strategy (please specify)

F. Additional considerations about the development of rapid reviews on diagnostic tests

Finally, it is of our interest to know some details about the final report of the findings and the completion of the process.

Select all the strategies used by your institution while conducting a rapid review of a diagnostic test:

- [ ] A brief template (less than 10 pages) is used to prepare the review report
- [ ] A peer-review process (external/internal) is performed
- [ ] A final assessment of the certainty of the evidence (under the GRADE approach) is performed
- [ ] A public consultation is open to collect comments about the review
- [ ] The final version of the review is considered for publication (including peer review journals)
- [ ] The fulfillment of the stakeholder request is revised after the end of the process

F. Additional considerations about the development of rapid reviews on diagnostic tests (continuation)

Are there other strategies, not previously mentioned, that your institution used whil[illegible] conducting a rapid review of a diagnostic test?

- ◯ No
- ◯ Yes (please specify)

Thank you for taking the time to participate in this survey

Survey responses will be de-identified; therefore, your name will not be linked to your survey responses. The results of this study may be presented at conferences, seminars or other public forums, and published in journals. You will not be identified individually, and all results will be presented in aggregate.

If you want to receive information about the results of this survey, please provide your email address

Email Address ____________________
